# The first case of huge amebic intra-abdominal tumor with asymptomatic amebic colitis

**DOI:** 10.1186/s40792-015-0053-1

**Published:** 2015-06-16

**Authors:** Shigeo Higami, Eiji Nomura, Masashi Yamazaki, Seiji Morita, Wataru Noguchi, Shuji Uda, Hitoshi Hara, Soichiro Yamamoto, Sayuri Hasegawa, Kosuke Tobita, Takuma Tajiri, Masaya Mukai, Sadaki Inokuchi, Hiroyasu Makuuchi

**Affiliations:** Department of Emergency and Critical Care, Tokai University Hachioji Hospital, 1838 Ishikawa-machi, Hachioji Tokyo, 192-0032 Japan; Department of Gastroenterological and General Surgery, Tokai University Hachioji Hospital, 1838 Ishikawa-machi, Hachioji Tokyo, 192-0032 Japan; Department of Emergency and Critical Care Medicine, Tokai University School of Medicine, 143 Shimokasuya, Isehara, Kanagawa 259-1193 Japan; Department of Pathology, Tokai University Hachioji Hospital, 1838 Ishikawa-machi, Hachioji Tokyo, 192-0032 Japan

**Keywords:** *Entamoeba histolytica*, Intra-abdominal tumor, Amebic colitis

## Abstract

We report a rare case of huge amebic intra-abdominal tumor with asymptomatic amebic colitis. This appears to represent the first report of amebic intra-abdominal tumor. A 31-year-old woman presented to a local doctor with only a sensation of abdominal fullness. Abdominal computed tomography (CT) showed a huge intra-abdominal tumor in the left abdominal cavity, and she was referred to our hospital. Colonofiberscopy for detailed examination showed multiple slight, discrete ulcers in the cecum. Ameboid trophozoites were identified from biopsy specimens, and asymptomatic amebic colitis was diagnosed. Oral metronidazole (MTZ) was administered at 1500 mg/day for 10 days. CT 14 days after starting MTZ showed no change in the intra-abdominal tumor, and resection of the tumor was therefore performed. Pathological examination revealed *Entamoeba histolytica* with engulfed erythrocytes complicated by hemorrhagic cyst. If an intra-abdominal tumor is present and colitis is observed, amebic intra-abdominal tumor should be considered among the differential diagnoses.

## Background

Infection by *Entamoeba histolytica* causes amebic colitis, which often presents as cramping abdominal pain and bloody diarrhea. While the majority of *E. histolytica* infections may be asymptomatic, approximately 10 % of amebic infections develop to invasive disease. Approximately 1 % of cases of invasive amebic disease develop extraintestinal disease through the bloodstream, affecting tissues such as the liver [1, 2], lungs [3–5], pericardium [6, 7], brain [8–10], and skin [11, 12]. Invasive *E. histolytica* infection may result in severe and potentially fatal illness, but no case reports appear to have described amebic intra-abdominal tumors.

## Case presentation

**Fig. 1:**
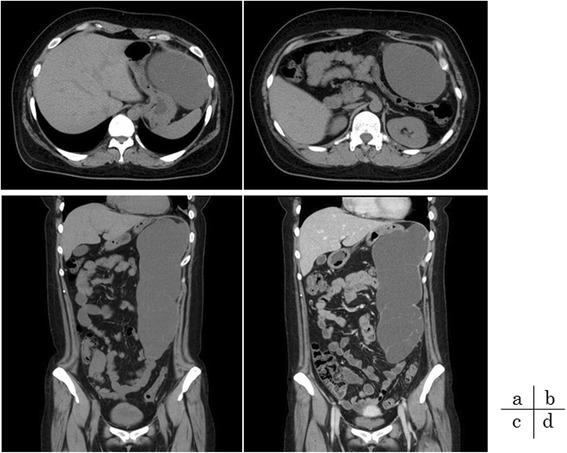
Findings of computed tomography (CT). **a**–**c** CT on the first visit to our hospital shows a multicystic tumor measuring 270 × 100 × 100 mm and continuous with the greater curvature of the stomach. **d** CT after 14 days of MTZ treatment reveals no change in size or structure, and no presence of nodules

**Fig. 2:**
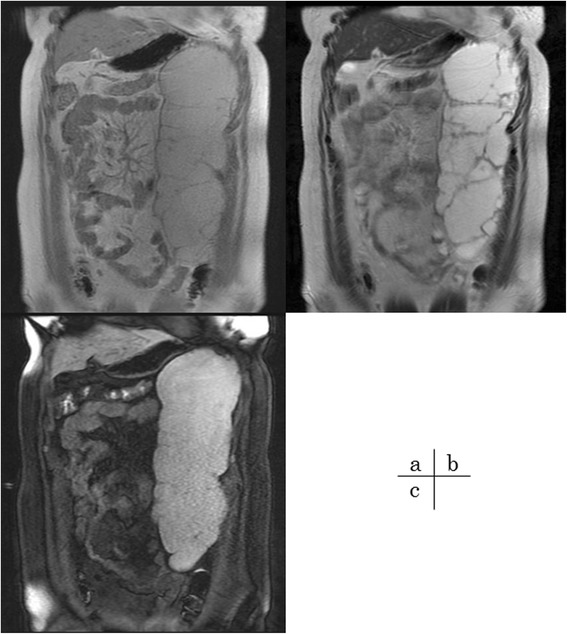
A hyperintense multicystic tumor on magnetic resonance imaging (MRI). **a** T1-weighted imaging. **b** T2-weighted imaging. **c** Fat-suppression T1-diffusion-weighted imaging. The tumor contains blood component and no fatty component

**Fig. 3:**
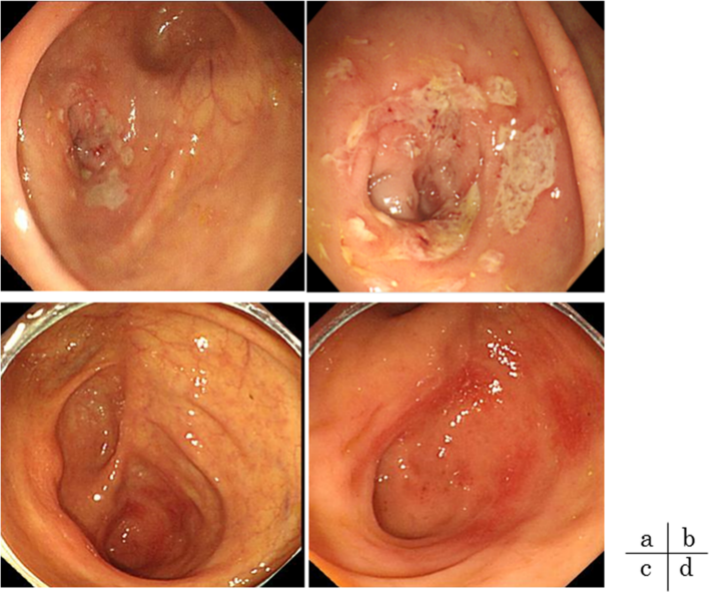
Colonofiberscopic findings. **a**, **b** Before MTZ administration. Multiple slight, discrete ulcers of different sizes are seen in the cecum around the appendiceal orifice. Ameboid trophozoites were evident in biopsy specimens. **c**, **d** After MTZ administration. The ulcers have improved, with only reddened mucosa seen after starting MTZ. Ameboid trophozoites were not evident in biopsy specimens

**Fig. 4:**
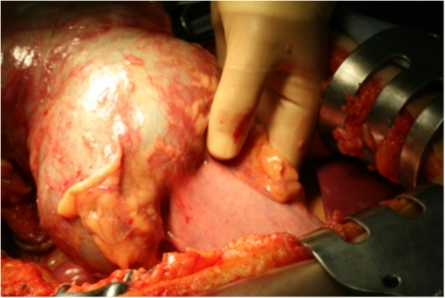
Operative findings. *Right side*, cranial; *left side*, caudal. The tumor is in the omental bursa, in contact with the greater curvature of the stomach. No adhesions with other adjacent organs are present. Tumor resection with partial gastrectomy was performed without bursting the tumor

**Fig. 5:**
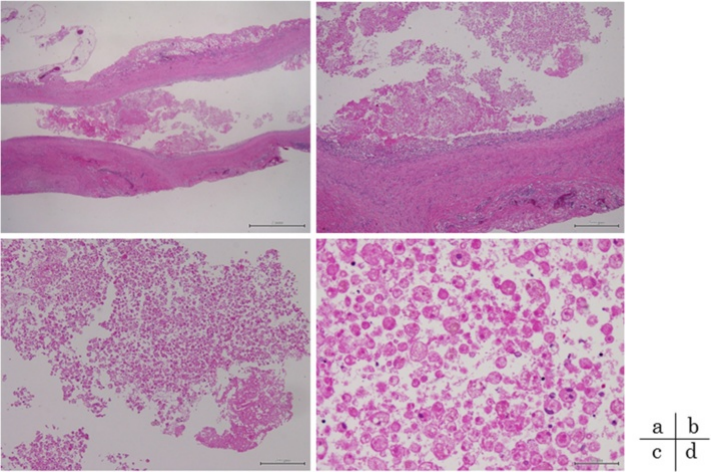
Pathological findings of the tumor (hematoxylin and eosin staining for **a**–**d**). **a**, **b** Deposition of hemosiderin and accumulation of macrophages including multinuclear giant cells in the cystic wall are evident. No endothelial cells are apparent in the cystic wall. The tumor does not show contact with the stomach on pathological examination. **c**, **d** The tumor shows trophozoites of *E. histolytica* engulfing erythrocytes

A 31-year-old woman presented to a local doctor complaining only of a sensation of abdominal fullness. Abdominal computed tomography (CT) showed a huge intra-abdominal tumor in the left abdominal cavity. She was referred to our hospital for detailed examination and treatment. Physical examination revealed a palpable, slightly hard mass with smooth surface in the left abdomen. The patient had never been overseas. Laboratory examination showed no marked abnormalities (white blood cells, 8700/mm^3^; C-reactive protein, 1.006 mg/dl). CT revealed a multicystic tumor measuring 270 × 100 × 120 mm, continuous with the greater curvature of the stomach (Fig. 1). Magnetic resonance imaging revealed a multicystic mass appearing hyperintense on T1-weighted imaging, T2-weighted imaging, and fat-suppression T1-diffusion-weighted imaging. The tumor contained blood component and no fatty component (Fig. 2). Colonofiberscopy for detailed examination showed multiple slight, discrete ulcers of different sizes in the cecum around the appendiceal orifice (Fig. 3). Ameboid trophozoites were found in the biopsy specimens. Based on a diagnosis of asymptomatic amebic colitis, metronidazole (MTZ) was orally administered at a dose of 1500 mg/day for 10 days. Colonofiberscopy performed 11 days after starting administration of MTZ showed only reddened mucosa with ulcer scars (Fig. 3), and ameboid trophozoites were not found in the biopsy specimens. However, CT after 14 days of MTZ showed no change in the size and structure of the tumor (Fig. 1), so surgery was performed. The tumor was located in the omental bursa, in contact with the greater curvature of the stomach. No adhesion with organs other than the stomach was evident. Tumor resection with partial gastrectomy was therefore performed in consideration of the primary tumor derived from the stomach, taking care not to rupture the tumor (Fig. 4). The resected tumor was 276 × 144 mm in size and weighed about 2800 g. Pathological findings (Fig. 5) revealed a multilocular cystic tumor due to infection by *E. histolytica*. Postoperative course was uneventful, and the patient was discharged from the hospital 9 days after surgery.

## Discussion

Amebiasis is a parasitic infection caused by the intestinal protozoan *E. histolytica*. This parasite is estimated to infect about 50 million cases annually, with about 40,000–100,000 deaths each year. *E. histolytica* infection occurs when mature cysts are ingested, typically through fecally contaminated water or food, most frequently in the developing world. *E. histolytica* infections are commonly observed in travelers, recent immigrants, homosexual men, and inmate populations. Pregnant women, individuals with diabetes, immunocompromised individuals, patients infected with human immunodeficiency virus, and those receiving corticosteroids are at particular risk of fulminant disease [13–17]. In Japan, *E. histolytica* is classified as a category V infectious disease according to the Infectious Diseases Control Law (concerning the prevention of infections and medical care for patients with infections). Doctors diagnosing *E. histolytica* are required to report the case to a public health department within 7 days of diagnosis. According to the report of the National Institute of Infectious Disease, the number of cases of *E. histolytica* infection has been increasing annually, particularly since 2000, and this has been attributed to an increase in sexually transmitted cases. The number of *E. histolytica* cases was reported as 1047 cases in 2013. The major reported cities in 2013 were Tokyo, with 188 cases, and Osaka, with 106 cases. Given that registration of *E. histolytica* cases is obligatory in Japan, the breakdown of causes of infection was reported in 2012 as unknown in 51.9 % and sexually transmitted in 34.1 % [18]. Because the patient had not visited an endemic area or traveled overseas, and reported no sexual contacts, the route of infection in this case was reported to the public health department as unknown.

Infection with *E. histolytica* is reportedly asymptomatic in approximately 90 % of cases. However, 4–10 % of initially asymptomatic individuals infected with *E. histolytica* develop symptomatic amebiasis over the course of the following year. *E. histolytica* can cause invasive intestinal and extraintestinal infections, which may occasionally result in severe and potentially fatal illness. The most common manifestation of invasive amebiasis is colitis, typically presenting as a several-week history of cramping abdominal pain, weight loss, and bloody diarrhea. Common sites of extraintestinal lesions are the liver and lungs, while rare case reports have described infection of the pericardium, brain, and skin. A search of PubMed using a combination of key words such as “*Entamoeba histolytica*”, “amebiasis,” and “intraabdominal tumor” through December 31, 2014 yielded no reports of amebic intra-abdominal tumor. Likewise, no mention of amebic intra-abdominal tumor was found in various reviews of *E. histolytica* infection [13–15, 19]. Amebic intra-abdominal tumor thus appears extremely rare.

In this case, diagnosis of the intra-abdominal tumor by imaging alone was very difficult. Differential diagnoses for intra-abdominal tumor include cystic mesothelioma, omental cyst, cystic lymphangioma, liposarcoma, and gastrointestinal stromal tumor. In these cases, surgical excision is usually indicated for diagnosis and treatment [20, 21].

Many reports have suggested that extraintestinal amebiasis spreads hematogenously [8, 22]. Shamsuzzam et al. reported that infection by thoracic amebiasis usually spreads by extension of an amebic liver abscess and also spreads hematogenously, lymphogenously, and by inhalation of dust containing cysts and trophozoites of *E. histolytica* [3]. In this case, we hypothesized that the intra-abdominal tumor developed by hematogenous spread from amebic colitis, although we could not pathologically confirm cysts or trophozoites in the small vessels or lymphatic vessels. If an intra-abdominal tumor was present and amebic colitis was observed, tumor related to *E. histolytica* would need to be considered among the differential diagnoses, and removal should be performed in such a manner as to avoid damaging the tumor.

In some reports and reviews, a typical treatment regimen for *E. histolytica* infection has been MTZ for 10–14 days (500–750 mg, three times/day) or tinidazole for 3 days (2 g/day), followed by a 7-day course of paromomycin (25–35 mg/kg daily in three divided doses) to eliminate colonization [13, 14, 19]. In contrast, Watanabe et al. reported that the cumulative probability of recurrent invasive amebic disease after MTZ or tinidazole treatment showed no significant difference with or without subsequent luminal treatment. Controversy remains regarding the need for cyst eradication using paromomycin following MTZ or tinidazole [23]. Although we did not administer paromomycin after MTZ treatment and surgery in this case, no recurrence of symptoms due to *E. histolytica* has been seen to date.

No standard treatment strategy for amebic intra-abdominal tumor has yet been established. We might have achieved cure with MTZ treatment alone, using the treatment strategy for amebic liver abscess [13, 14, 22, 24, 25], but we considered complete resection of the tumor as prudent for this case in which definitive diagnosis had not been achieved. Either way, close attention must be paid to avoid bursting the cystic tumor at the time of extraction.

## Conclusions

Intra-abdominal tumor may represent asymptomatic *E. histolytica* infection and warrants colonofiberscopy. When amebic colitis is observed, amebic intra-abdominal tumor should be considered as a differential diagnosis.

## Consent

Written informed consent for publication of this case report and the accompanying images was obtained from the patient. A copy of the written consent is available for review by the Editor in Chief of this journal.
